# *Pseudozyma* Priming Influences Expression of Genes Involved in Metabolic Pathways and Immunity in Zebrafish Larvae

**DOI:** 10.3389/fimmu.2020.00978

**Published:** 2020-05-22

**Authors:** Prabhugouda Siriyappagouder, Jorge Galindo-Villegas, Anusha K. S. Dhanasiri, Qirui Zhang, Victoriano Mulero, Viswanath Kiron, Jorge M. O. Fernandes

**Affiliations:** ^1^Faculty of Biosciences and Aquaculture, Nord University, Bodø, Norway; ^2^Department of Cell Biology and Histology, Faculty of Biology, Institute of Biomedical Research of Murcia-Arrixaca, Campus Universitario de Espinardo, University of Murcia, Murcia, Spain

**Keywords:** immune system, commensal yeast, germ-free, transcriptome, *Danio rerio*, *Pseudozyma* sp.

## Abstract

Fungi, particularly yeasts, are known essential components of the host microbiota but their functional relevance in development of immunity and physiological processes of fish remains to be elucidated. In this study, we used a transcriptomic approach and a germ-free (GF) fish model to determine the response of newly hatched zebrafish larvae after 24 h exposure to *Pseudozyma* sp. when compared to conventionally-raised (CR) larvae. We observed 59 differentially expressed genes in *Pseudozyma*-exposed GF zebrafish larvae compared to their naïve control siblings. Surprisingly, in CR larvae, there was not a clear transcriptome difference between *Pseudozyma*-exposed and control larvae. Differentially expressed genes in GF larvae were involved in host metabolic pathways, mainly peroxisome proliferator-activated receptors, steroid hormone biosynthesis, drug metabolism and bile acid biosynthesis. We also observed a significant change in the transcript levels of immune-related genes, namely *complement component 3a, galectin 2b, ubiquitin specific peptidase 21*, and *aquaporins*. Nevertheless, we did not observe any significant response at the cellular level, since there were no differences between neutrophil migration or proliferation between control and yeast-exposed GF larvae. Our findings reveal that exposure to *Pseudozyma* sp. may affect metabolic pathways and immune-related processes in germ-free zebrafish, suggesting that commensal yeast likely play a significant part in the early development of fish larvae.

## Introduction

Germ-free (GF) organisms are ideal research models to reveal the effects of exposure to either one selected microbial species or microbial consortia from a particular host. Even if fungi account for only a small proportion of the vertebrate microbiota, they play a significant role in shaping the microbial community structure, metabolic functions, and modulation of the host immune responses (1). Fungi are particularly well known for their involvement in immunomodulatory processes and their health inducing properties (2, 3). Accumulating evidence indicates that yeasts, its metabolites and cell wall components are key players in the physiology of all hosts, including fish (4, 5). Until recently, only few yeast strains/species like *Saccharomyces cerevisiae, Debaryomyces hansenii* and *Candida albicans* were exploited for industrial and commercial purposes (5–7). However, studies have demonstrated the protective functions of non-Saccharomycetaceae species like *Yarrowia lypolitica* 242 (Yl242) in zebrafish (*Danio rerio*) against *Vibrio anguillarum* infection (8). In addition, the extremophile strain *Sterigmatomyces halophilus* N16 has been labeled as a novel fish immunostimulant due to its beneficial effects on the growth and immune response of (*Sparus aurata*) gilthead seabream (9).

Comparison of GF mice with those colonized with normal microbiota has demonstrated that microbiota-induced alterations of gene expression are associated with immune homeostasis and are essential for ideal metabolic orientation of the host (10, 11). Furthermore, colonization of GF mice with *Bacteroides thetaiotaomicron* and *Bifidobacterium longum* has altered the metabolic processes (12). Similarly, exposure of GF mice to the same bacterial ecosystem as those of conventionally raised mice altered the lipid absorption-linked hepatic metabolites, which were found to interact with specific bacteria (13). Another study in GF mice has demonstrated the importance of commensal yeasts in regulating metabolic pathways and affecting the expression of genes involved in intestinal barrier maintenance (14). DNA microarray gene expression profiling of GF zebrafish larvae colonized with gut microbiota has provided the possibility to understand the role of bacteria in stimulating epithelial proliferation, nutrient metabolism and innate immune response (15). In addition, it has been shown that early exposure of zebrafish larvae to commensal microbes can prime neutrophils, induce proinflammatory and antiviral genes and increase the resistance to viral infection (16). In our previous study, we have shown that exposure to *Pseudozyma* sp. or *Debaryomyces* sp. during early ontogeny can remarkably alter the intestinal bacterial composition of zebrafish larvae (17). *Pseudozyma* spp. are yeast-like fungi, related to the Ustilaginales order and belonging to the phylum Basidiomycota. Some species of *Pseudozyma* are known to secrete extracellular metabolites and biosurfactants with great potential for biocontrol applications (18–20). The cell wall of these yeasts consists mainly of β-glucans (21) with immunomodulatory properties considered beneficial to fish health (22).

Thus, far, the influence of yeast on host transcriptomic responses has not been reported in fish. In order to elucidate how *Pseudozyma* sp. contact with external tissues and mucosal surfaces (excluding intestinal tissues) during early ontogeny influences metabolic and immunity-related pathways, we performed RNA-sequencing to analyze the transcriptomic response of germ-free (GF) and conventionally raised (CR) zebrafish larvae upon *Pseudozyma* exposure.

## Materials and Methods

### Ethics Statement

All experimental procedures involving zebrafish were carried out in compliance with the Guidelines of the European Union Council (Directive 2010/63/EU) and the Spanish RD 53/2013. Experiments that were conducted in Spain, were approved by the Bioethical Committee of the University of Murcia (licenses #537/2011, #75/2014, and #216/2014).

### *Pseudozyma* sp. and Culture Conditions

The yeast strain used in this study was originally isolated from the intestine of zebrafish (AB strain) that were reared in a recirculating system (Pentair Aquatic Eco-Systems, NC, USA) at Nord University, Bodø. The isolated yeast colonies were identified by PCR amplification and Sanger sequencing of the internal transcribed spacer 2 (ITS2) region of the fungal rDNA, using fITS7 (GTGARTCATCGAATCTTTG) and ITS4 (TCCTCCGCTTATTGATATGC) primers (23, 24). Identification at the species level was determined by BLASTN similarity searches against the National Centre for Biotechnology Information (NCBI) GenBank database using default parameters. Pure yeast cultures were prepared and stored in 30% (v/v) glycerol (Sigma-Aldrich, St. Louis, MO, United States) at −80°C. Prior to use in the exposure studies, the cultures were revived on yeast extract peptone dextrose agar (Sigma-Aldrich) plate and broth supplemented with 0.025% chloramphenicol (Sigma-Aldrich). A single colony from the agar plate was inoculated and further grown in yeast extract peptone dextrose broth at 28°C, shaking the culture flasks at 180–200 rpm for 24 h. The cultured yeast cells were then harvested by centrifugation at 10,000 rpm for 10 min, washed and resuspended in sterile phosphate-buffered saline (PBS, Sigma-Aldrich) to a final concentration of 2 × 10^5^ CFU/ml for the exposure study.

### Zebrafish Husbandry and Preparation of Larvae

Standard husbandry procedures (25) were followed to maintain wild-type (AB strain) and Tg(*mpx::eGFP*)^*i*114^ zebrafish in a re-circulation system (Aqua Medic GmBH, Bissendorf, Germany) at the Department of Cell Biology and Histology, University of Murcia. Germ-free (GF) and conventionally-raised (CR) zebrafish larvae for the exposure experiment were generated by following the method described by Pham et al. (26) with slight modification, as previously detailed in Siriyappagouder et al. (17).

### Yeast Exposure Study

Zebrafish larvae, both GF and CR, were aseptically divided into two groups (maintained in triplicate cell culture flasks): control (PBS) and yeast-exposed (*Pseudozyma*). Each group consisted of 60 larvae randomly distributed into the 25 mL rearing flasks, each holding 20 larvae. The different study groups and their abbreviations are as follows: conventionally-raised control (CRC), conventionally-raised yeast-exposed (CRY), germ-free control (GFC), and germ-free yeast-exposed (GFY). At 2 days post-fertilization (dpf), GF and CR zebrafish larvae were exposed to 2 × 10^5^ CFU/ml *Pseudozyma* sp. and incubated for 24 h (until 3 dpf) at 28°C. On the other hand, the control larvae were exposed to PBS. After the 24 h exposure, zebrafish larvae were washed (3×) with regular embryo medium and euthanized with an overdose of MS222 (Sigma-Aldrich, Madrid, Spain). Nine pools of five larvae per treatment group were collected in screw cap tubes on dry ice and immediately stored at −80°C for later use.

### Live Imaging of Neutrophils

At 72 hpf, larvae of the transgenic line Tg(*mpx::eGFP*)^*i*114^ in which the GFP expression is driven by the myeloid-specific peroxidase (*mpx*) promoter and whose neutrophils are green fluorescent (27), were anesthetized with tricaine methanesulfonate (200 mg/L, Sigma-Aldrich) and mounted in 1% (w/v) low melting point agarose (Sigma-Aldrich) dissolved in egg water. Fish were imaged with an epifluorescence stereomicroscope LeicaMZ16F (Leica, Germany) equipped with green fluorescent filters, while they were kept in their agar matrices with the medium at 28.5°C (16). High-quality images were subsequently used to determine the number of fluorescent neutrophils (*mpx*::eGFP) present at the caudal hematopoietic tissue (CHT) in each fish per condition using the Cell Counter plugin in ImageJ (https://imagej.nih.gov/ij/plugins/cell-counter.html).

### RNA Isolation, Library Preparation and mRNA Sequencing

Total RNA was extracted from pooled larvae following the QIAzol protocol (Qiagen, Hilden, Germany). RNA quality, purity and quantity were determined using the NanoDrop 1000 (Thermo Fisher Scientific, Waltham, MA, United States). Furthermore, RNA integrity was assessed using Agilent RNA high sensitivity screen tape kits on the 2200 TapeStation system (Agilent Technologies, Santa Clara, CA, USA). Only samples with RIN ≥ 7 were used for library preparation.

RNA-seq libraries were prepared using the NEBNext ultra II directional RNA library kit with the poly (A) mRNA magnetic isolation module (NEB #E7490), according to the manufacturer's protocol (New England, BioLabs Inc. UK). In brief, one microgram of total RNA was used for library preparation and mRNA was enriched using oligo-dT magnetic beads and fragmented to ~100–200 nt prior to synthesis of the first and second cDNA strands. The resulting cDNA was purified and the 3′ end was repaired for adapter ligation. PCR enrichment (eight cycle) was performed and the amplified libraries were cleaned with AMPure XP beads (Beckman Coulter, Inc. Brea, CA, United States) to ensure that they were free from residual adapter dimers and unwanted (smaller) fragments. In total, 16 libraries were prepared, i.e., four replicates per treatment group. Individual libraries were quantified, normalized, pooled at equimolar ratio, and sequenced as single-end reads (75 bp) on the Illumina NextSeq 500 sequencer (Illumina, San Diego, CA, United States) with NextSeq 500/550 high output v2 kit.

### Bioinformatic Analysis of Differential Gene Expression

Adapter sequences were removed from the raw reads using cutadapt with the following parameters: -q 25, 20 –quality-base = 33 –trim-n -m 20 (28). Quality of clean reads was further assessed using FastQC (29) and reads with quality <30 were removed. Bowtie 2 (30) and Tophat2 were used to build the index and align the reads. Cleaned reads were mapped to the zebrafish transcriptome (GRCz10.86.chr.gtf) and genome (GRCz10.dna.toplevel.fa) from Ensembl (http://www.ensembl.org) with TopHat2, version 2.1.0 (31). Gene expression values were computed by HTSeq (http://htseq.readthedocs.io/en/release_0.9.1/). DESeq2 package (32) was used to determine the differentially expressed genes; transcripts with more than 1.5-fold change in the treatment groups compared to the control group, and adjusted *p* < 0.05 (Benjamini–Hochberg multiple test correction method) were considered significantly different, and they were used for downstream analysis. Kyoto Encyclopedia of Genes and Genomes (KEGG) pathway enrichment was studied using clusterProfiler (33). Transcriptome data were visualized using the R packages ggplot2 and pheatmap. The datasets generated in this study can be found in the Sequence Read Archive (NCBI) under the accession number PRJNA579488.

### Validation of RNA-Seq Data by Droplet Digital PCR (ddPCR)

We selected 5 genes, *fabp2, lgals2b, cyp7a1, aqp8a.2*, and *c3a.2* from the list of top DEGs (Supplementary Table 1) for validation of the RNA-Seq data. Primers for the target genes were designed using NCBI Primer BLAST and NetPrimer (Table 1). The primers for the reference genes (*eef1a* and *actb*) had been previously reported (37). One microgram of total RNA from each sample, GFY (*n* = 4) and GFC (*n* = 4) was reverse transcribed using the reverse QuantiTect transcription kit (Qiagen), according to the manufacturer's protocol. The obtained cDNA was further diluted 10 times with nuclease free water and used as PCR template. ddPCR was performed on a QX200™ Droplet Digital™ PCR System (Bio-Rad Laboratories, Inc., Hercules, CA, USA). In brief, the ddPCR reaction mixture consisting of 10 μl of QX200™ ddPCR EvaGreen Supermix (Bio-Rad), 1 μl of gene-specific primer pair, 4 μl of the cDNA sample was made up to a total volume of 20 μl with 5 μl of nuclease-free water. Purified distilled water was used as no template control. Droplets were prepared using a droplet generator with 70 μL of droplet generating oil and 20 μL of ddPCR reaction mixture. The thermocycling reaction was performed in a C1000 thermal cycler (Bio-Rad) with the following conditions: initial denaturation at 95°C for 10 min, followed by 40 cycles of 94°C for 30 s, 60°C for 1 min and a final elongation at 98°C for 10 min with 2°C/s ramp rate. The fluorescence intensity of each droplet was measured on the QX200 Droplet Reader (Bio-Rad). Positive and negative droplet cluster signals were manually separated by setting the threshold level of each gene based on the fluorescence level of the negative control. Relative expression of selected genes was determined based on the geometric mean of reference genes (*eef1a* and *actb*). After confirming that the data complied with normality (Shapiro-Wilk) and equal variance (F-test) assumptions, the Welch two sample *t*-test was used to detect significant differences between GFC and GFY groups.

**Table 1:** Details of the primers used for ddPCR.

**Gene**	**Forward primer (5′-3′)**	**Reverse primer (5′-3′)**	**Amplicon (bp)**	**Accession or references**
*fabp2*	TGGAACAAATGGGCGTCAAC	GTGCGGAAAGTGCTGACTTC	118	NM_131431
*lgals2b*	TGGCTGTGATGCCTTCTC	TGCTTTGAGTTGCAGACG	116	NM_200514
*cyp7a1*	TCTGCGAAGGCATTTGGTCA	GCCCTGGAGGTTTTCCATCA	141	NM_201173
*aqp8a.2*	CTAGCAGGAGGAGATGTTTC	CCAACGGACCGACCCAATA	112	NM_001080182
*c3a.2*	GCGGGGAAACACAGTCAGG	GCCATCAGAACATACAGCG	100	NM_131243
*il1b*	TGGACTTCGCAGCACAAAATG	GTTCACTTCACGCTCTTGGATG	150	(34)
*tnfα*	GCGCTTTTCTGAATCCTACG	TGCCCAGTCTGTCTCCTTCT	148	(35)
*il10*	TCACGTCATGAACGAGATCC	CCTCTTGCATTTCACCATATCC	151	(36)

*Gene names, primer sequences, amplicon sizes, NCBI accession numbers or references (in the case of published primers) are indicated*.

## Results

### RNA Sequencing Data Quality and Statistics

After removing one biological replicate from the CRY group due to the low number of raw reads (<5 million reads), a total of over 485 million sequencing raw reads were obtained from the remaining 15 samples. Following adapter trimming and quality filtering, we obtained 476 million reads (98%) that had a quality score of Q ≥ 30, of which over 433 million were mapped to zebrafish transcriptome and genome (Table 2). On an average over 90% of the filtered reads were mapped to zebrafish transcriptome and genome.

**Table 2:** Summary of RNA-seq sequencing and mapping statistics, Germ-free yeast-exposed (GFY 1–4), Germ-free control (GFC 1–4), conventionally-raised yeast-exposed (CRY 1–3) and conventionally-raised control (CRC 1–4).

**Groups**	**Sample Name**	**Raw reads**	**Trimmed reads**	**Mapped reads**
GFY	GFY1	24669117	24048794	21938786
	GFY2	37114905	36815478	33370813
	GFY3	21328279	20761050	18698371
	GFY4	31457716	31402738	28591929
GFC	GFC1	52777971	52741317	47873587
	GFC2	44926999	43756660	40008298
	GFC3	48821732	47864789	43618137
	GFC4	31078486	27438703	24860054
CRY	CRY1	19547159	19016863	17297348
	CRY2	20919983	20764342	18831659
	CRY3	18521634	18291999	17010316
CRC	CRC1	48588243	48529870	44062230
	CRC2	21689356	21011187	19126449
	CRC3	24736302	24621065	22202107
	CRC4	39589730	39389063	35532867
Total		485767612	476453918	433022951
		**Minimum**	**Maximum**	**Total**
	Raw reads	18521634	52777971	485767612
	Trimmed reads	18291999	52741317	476453918
	Mapped reads	17010316	47873587	433022951

### Differentially Expressed Genes

DEG analysis was performed by DESeq2 and the genes that had a fold change of 1.5 or more and with an adjusted *p* < 0.05 (Benjamin-Hochberg method) were included in the downstream analysis. We found 59 differentially expressed genes in GFY larvae compared with the GFC group. Of these, 57 genes were up-regulated and only 2 genes were down-regulated; these genes are listed in Supplementary Table 1. Two genes, namely *ankyrin repeat domain 37* (*ankrd37*) and *egl-9 family hypoxia-inducible factor* (*egln3)* were significantly downregulated (1.5-fold change) in GFY larvae compared to the control group. Of the 57-up-regulated genes, 11 (21%) genes whose sequences are not functionally characterized will be hereafter referred as “unknown” genes.

Several genes encoding proteins involved in metabolic functions were up-regulated (with similar fold-changes in the range 1.5–1.6). They included mainly *fabp2 protein* (*fabp2), tryptophan 2,3-dioxygenase A (tdo2a), steroid-5-alpha-reductase, alpha polypeptide 2a (srd5a2a)* and cytochrome P450 family proteins encoding genes *cyp7a1, cyp8b1, cyp2r1*, and *cyp2p8*. Members of solute-carrier family, which are associated with cellular homeostasis, were also up-regulated 1.5 fold: *slc26a3.2, slc24a2*, and *slc38a11*. Surprisingly, transcript levels of some genes involved in the phototransduction were up-regulated (1.5–1.8 fold), namely *phosphodiesterase 6H* (*pde6h*), *opsin 1 long-wave-sensitive-1* (*opn1lw1*), *opsin 1 short-wave-sensitive*-1(*opn1sw1*), *G protein-coupled receptor kinase 7 a* (*grk7a*), *rhodopsin* (*rho*), and *peripherin 2a* and *2b* (*prph2a, prph2b*). Furthermore, genes involved in immunity, including *complement component* (*c3a*), *galectin* (*lgals2b*), *ubiquitin specific peptidase 21* (*usp21), keratin, type 1, gene 19d* (*krt1-19d*), and *aquaporins* (*aqp8a* and *aqp9b*) were upregulated in response to yeast exposure. *Complement component c3a* was upregulated 1.8-fold while the other immune-related genes had a 1.5–1.6 fold increase. Surprisingly, comparison of the CRY and CRC larvae did not yield any differentially expressed genes.

**Figure 1:**
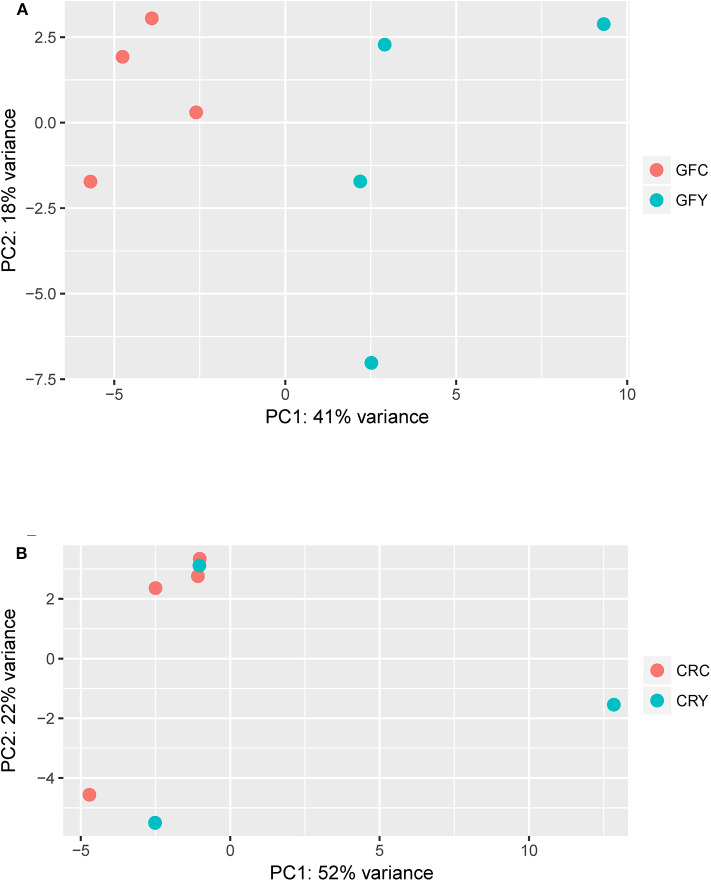
Principal component analysis plots showing the distinction between **(A)** GFY vs. GFC and **(B)** CRY vs. CRC. Except CRY, all groups comprise four biological replicates. CRC, Conventionally-raised control; CRY, conventionally-raised yeast-exposed; GFC, germ-free control; GFY, germ-free yeast-exposed.

**Figure 2:**
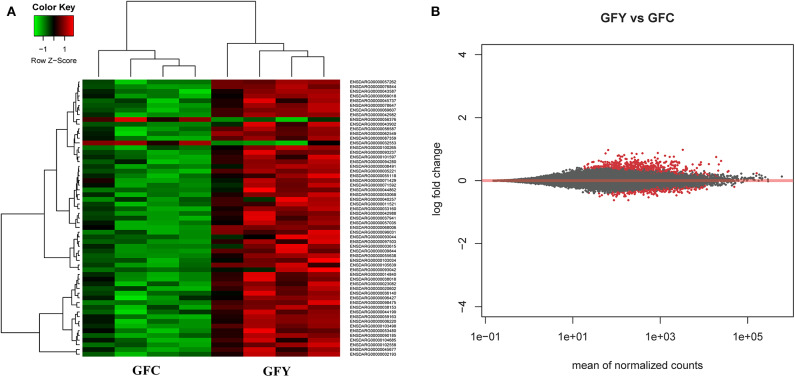
Heat map **(A)** and MA plot **(B)** of differentially expressed genes in GFY (gfy 1–4) compared to GFC (gfc 1–4). Each group comprise four biological replicates. The X-axis and Y-axis in the MA plot denote the mean normalized counts and log2 fold changes between the two groups, respectively. All differentially expressed genes with adjusted *p*-value below 0.05 are shown in red. The heat map only includes transcripts with |fold-change| > 1.5. GFC, Germ-free control and GFY, germ-free yeast-exposed.

The principal component analysis (PCA) plot illustrates that there was a clear separation of GFY compared with the GFC larvae (Figure 1A). The first principal component (41.0%) separates the yeast-exposed and control larvae, indicating that the 2 groups are clearly different. However, the second component was able to capture only 18% of the variation in the data and the component did not distinguish the 2 groups. Regarding the CR groups, CRY and CRC larvae were not distinctly clustered (Figure 1B), suggesting that they had similar transcriptomes. It also indicates a negligible transcriptomic difference between the groups but substantial intra-group variability. Hierarchical cluster analysis was also performed to understand the difference in the DEGs profile between the GFY and GFC. The yeast-exposed larvae and the control larvae clustered separately (Figure 2A). The differences in magnitude of the differentially expressed genes between groups are also illustrated in the MA plot (Figure 2B).

### Pathway Enrichment Analysis of DEGs Upon Yeast Exposure

**Figure 3:**
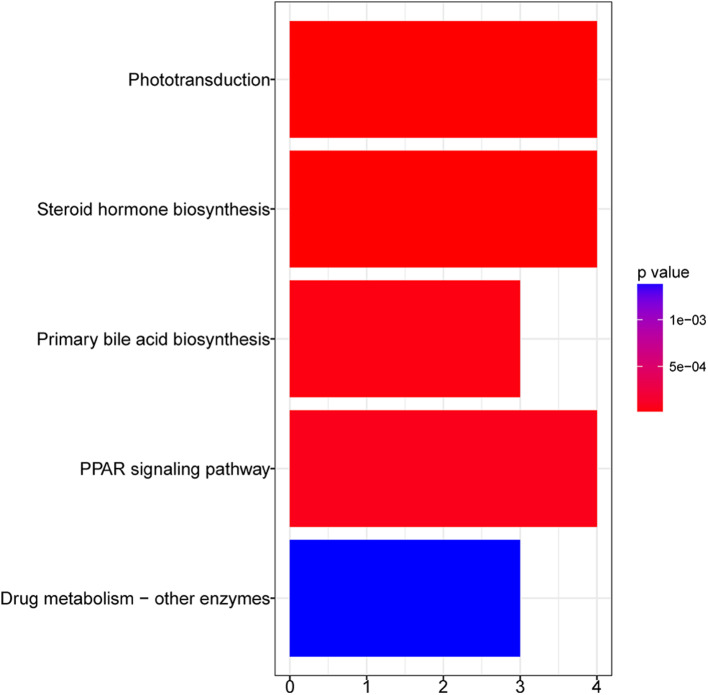
Bar plot of enriched KEGG pathways in GFY compared to GFC. The vertical-axis indicates the pathway name and horizontal-axis indicates the gene count. Significance was set at adjusted *p*-value < 0.05 (Benjamin-Hochberg method). GFC, Germ-free control and GFY, germ-free yeast-exposed.

Figure 3 shows the KEGG pathways that were significantly enriched in GFY larvae compared to the GFC group. Peroxisome proliferator-activated receptor (PPAR) was the most significantly enriched pathway, followed by steroid hormone biosynthesis and phototransduction pathways. Furthermore, cell metabolism-related pathways—including drug (xenobiotic) metabolism—other enzymes and primary bile acid biosynthesis pathways were also enriched. We did not obtain any enriched pathways for the downregulated DEGs in GFY when compared to GFC.

### Validation of Transcriptomic Data

**Figure 4:**
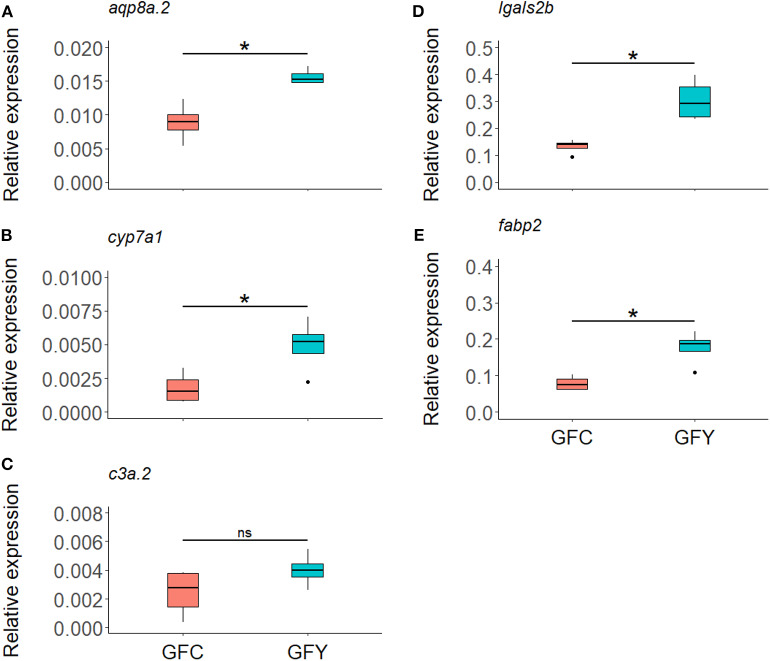
Relative expression of selected genes *aqp8a.2* **(A)**, *cyp7a1* **(B)**, *c3a.2* **(C)**, *lgals2b* **(D)**, and *fabp2* **(E)**, in germ-free control (GFC) and yeast-exposed (GFY) zebrafish larvae, as determined by ddPCR. The box plots show the median and 25th and 75th percentiles, based on four biological replicates. Asterisks denote significant differences, according to Welch two sample *t*-test.

The relative expression profiles of selected genes in GFC and GFY are shown in Figure 4. In yeast-exposed GF larvae, *fabp2, lgals2b, cyp7a1*, and *aqp8a.2* were significantly up-regulated by 125, 130, 180, and 75%, compared to the respective controls. The relative transcript levels of *c3a.2* were not significantly different between GFY and GFC fish but they were up-regulated with a 1.64-fold change following yeast exposure, as determined by RNA-seq analysis. Overall, the ddPCR results were in agreement with the transcriptomic data.

### Neutrophil Behavior After Yeast Priming (i.e., Exposure)

**Figure 5:**
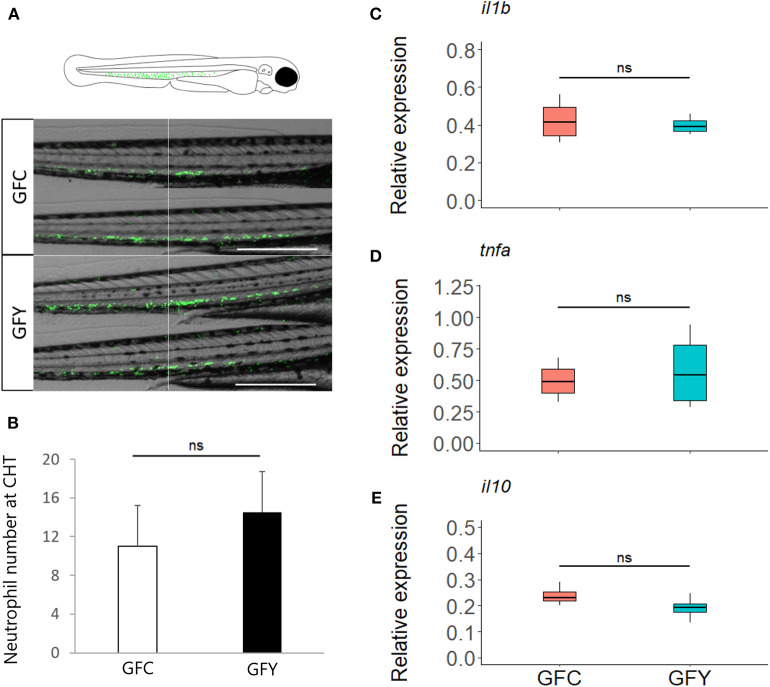
**(A)** Representative images of green fluorescent neutrophils at 3 days post-fertilization in Tg (*mpx*::eGFP)^i114^ germ-free control (GFC) and yeast-exposed (GFY) zebrafish larvae analyzed in triplicate. Scale bar, 200 μm. An augmented projection of the neutrophil portion located at the caudal hematopoietic tissue (CHT) is represented by the green area in the top drawing. **(B)** The neutrophil abundance between GFC and GFY at the caudal-hematopoietic tissue (CHT), *n* = 10, *P*-values were calculated with unequal variances *t*-test. **(C–E)** Relative expression of pro- (*ilib, tnf*α) and anti- (*il10*) inflammatory genes in GFC and GFY zebrafish larvae, as determined by ddPCR. The box plots show the median and 25th and 75th percentiles, based on four biological replicates. “n.s.” denotes non-significant differences between the groups.

Real-time imaging showed that contact with the commensal yeast *Pseudozyma* sp. for 24 h after hatching does not induce neutrophils to a pro-inflammatory state. Neutrophil distribution at the caudal hematopoietic tissue of fluorescent *mp*x reporter GF zebrafish larvae (3 dpf) in GFC vs. GFY is shown in Figure 5A (GFC vs. GFY), and the average neutrophil number per fish larvae is provided in Figure 5B. Both neutrophil distribution and number did not change significantly with yeast exposure. We found that the relative expression profiles of the key pro-inflammatory (*il1b* and *tnf*α), and anti-inflammatory (*il10*) marker genes did not change between both conditions tested (Figures 5C–E).

## Discussion

Host-associated bacteria are known to modulate metabolic and immune-related pathways that affect host physiology and overall development in mammals (12). Furthermore, early exposure to microbiota is essential for the development of specific immune components and their long-lasting effects on the host physiology (38). Previous studies have demonstrated the competence of commensal microbiota to prime the host immune responses (16, 39). However, the effect of early yeast exposure on the host transcriptome and immune development during early developmental stages of fish has received scant attention. Here we provide insight into differentially regulated genes and pathways in GF zebrafish larvae. The transcriptomic changes were associated with a variety of physiological processes, mainly steroid hormone biosynthesis, drug or xenobiotic metabolism, bile acid biosynthesis pathways (Figure 3) and some immune-related genes. Surprisingly, *Pseudozyma* exposure did not have any major effect on the transcriptome of CR zebrafish larvae. Taken together, our data demonstrate the ability of *Pseudozyma* sp. to modulate the transcriptome and shape metabolic processes but without inducing an inflammatory response in GF larvae. Quantification of transcript levels of selected genes by ddPCR and the real-time live imaging of neutrophils confirmed the transcriptomic results.

Cytochrome P450 represents a large family of enzymes that oxidize many xenobiotics and endogenous compounds, including therapeutic drugs, vitamins, steroids, fatty acids and environmental toxicants (40). *Pseudozyma* sp. exposure upregulated genes belonging to the cytochrome P450 family, namely *cyp7a1, cyp8b1, cyp2r1*, and *cyp2p8*. Among these, *cyp7a1* and *cyp8b1* encode key enzymes associated with bile acid synthesis (41). Previous studies in GF mice showed that exposure to bacteria can also have an impact on bile acid synthesis (42). The bacterial colonization process stimulated glycogenesis and increased triglyceride synthesis in liver by altering *cyp8b1* expression, thus modifying bile acid metabolism (13).

Commensal yeasts isolated from the fish intestine produce extracellular enzymes (43), which are most likely to have the ability to metabolize xenobiotics similar to those of gut bacteria (44). *Pseudozyma* sp. is known to produce various bio-active compounds, including several polyunsaturated fatty acids, glycolipids and squalene (45). Many of these bio-active compounds have antifungal or biosurfactant properties with full therapeutic effects and possible pharmacological applications, like squalene (46). Therefore, the activation of the drug (xenobiotic) metabolism pathway might be related minimizing the undesired toxic effect of secreted compounds from the yeast. The expression of mammalian homologs *cyp1a1, cyp2b6*, and *cyp2c19*, which are linked to xenobiotic metabolism, were modulated in the intestine of GF zebrafish colonized with zebrafish microbiota (15).

Upregulation of genes such as *cyp2r1* and *cyp7a1*, which encode cytochrome P450 enzymes, and *srd5a2a* (steroid-5-alpha-reductase, alpha polypeptide 2a) are associated with steroid metabolism (47). Implication of steroid hormones on embryonic development of zebrafish is poorly studied (48). However, a study performed on *Drosophila* has demonstrated the importance of steroid hormones in the regulation of innate immune function (49).

The present study was performed using 3 dpf zebrafish larvae, i.e., before their intestinal tract is fully developed. The effect of yeast exposure on the transcriptome of CR zebrafish larvae was not evident, possibly due to already existing xenobiotic-metabolizing microbes in the larvae. Alternatively, bacteria associated with CR zebrafish might have inhibited the influence of yeast, similarly to commensal bacteria of mice that are known to inhibit colonization by *C. albicans* (50).

Microbiota or their metabolites can act as ligands and modulate the host metabolic pathways via activation of nuclear receptors signaling. Peroxisome proliferator-activated receptors (PPARs) are members of nuclear receptor family that are activated by steroid hormones, fatty acids and their derivatives (51). PPARs mediate various cellular processes, and they influence lipid metabolism and modulate inflammatory responses (52). The yeast *Pseudozyma* sp. produces large amounts of squalene and several polyunsaturated fatty acids (45). Hence, it is likely that these fatty acids or other metabolites secreted by *Pseudozyma* could bind to fatty acid binding proteins (*fabp2*) and initiate the PPAR signaling pathway. In addition, *cyp7a1, cyp8b1* are also involved in the PPAR pathway. A previous study reported that *S. cerevisiae var. boulardii* can regulate the expression of—PPAR-γ in human colonocytes and reduce its response to pro-inflammatory cytokines (53). Furthermore, PPAR-γ maintains the oxygen levels in the gut lumen to help the growth of beneficial microbes and stave off the growth of pathogens (54).

Galectins belong to the evolutionarily conserved family of β-galactoside-binding proteins and they are known to be involved in a wide range of biological activities including regulation of immune function (55, 56). For, instance, Linden et al. (57) have appreciated the involvement of *galectin 3* in murine defense against systemic infection with *Candida* spp. In the present study, we have observed induction of *galectin 2* in response to yeast exposure. It indicates the potential role of this protein in immune modulation by binding to the β-galactoside, a crucial cell wall component of fungi (58).

Mannose-binding lectin, a pattern recognition molecule, binds to mannose and N-acetylglucosamine structure of fungi. It has been reported that this molecule plays a decisive role during yeast infection; by enhancing complement activation and increasing the uptake by polymorphonuclear cells (59). *Complement component 3a* is the central component of the complement system, which mediates the killing/blocking of pathogens during early developmental stages of fishes (60, 61). Furthermore, previous studies have reported the pivotal role of complement components during antifungal defense (62, 63). Among the up-regulated immune genes, *c3a* transcript levels had a higher fold change compared to other immune-related genes. Our results are in line with a previous study that demonstrated that *c3* is upregulated in GF zebrafish larvae treated with fish-derived yeast, either *D. hansenii*, or *Y. lipolytica* compared to non-treated GF larvae (8). Furthermore, orally delivered purified yeast beta-glucan upregulated *cr3* expression in the intestine of Atlantic salmon (64).

Ubiquitin and ubiquitin-like proteins are involved in spatial and/or temporal modulations of several immune signaling pathways (65), including those associated with C-type lectin receptors (CLRs) that regulate the antifungal immune response (66). For example, deficiency of E3 ubiquitin ligase CBLB in mice decreases activated CLR degradation, which in turn increases the expression of proinflammatory cytokines and enhances the inflammatory responses against fungi *C. albicans* (67, 68). We noted the upregulation of *usp21*, which modulates the innate immune responses through inhibition of the NF-κB-signaling cascade, which leads to down-regulation of TNFα (69). Hence, upregulation of *usp21* in yeast-exposed larvae could be attributed to regulation of immune response.

Aquaporins are membrane channel proteins that enable the host to regulate their cell volume and shape to deal with external and internal stimuli. These proteins are involved in various biological processes including inflammation and migration of immune cells (e.g., neutrophils) during exposure to microbes (70, 71). It has been reported that feeding Atlantic salmon with a *Candida utilis*-supplemented diet increases the expression of aquaporin-8ab (*aqp8ab*) and modulates the expression other immune-related genes, indicating that inclusion of yeast in fish diets may enhance their performance and health status (72). The differential expression of aquaporins *aqp8a* and *aqp9b* in our study point to their possible role in modulating the immune response upon yeast exposure.

The increased expression of genes encoding proteins involved in phototransduction pathways was surprising. It could be related to a technical problem during sampling, abundance of yeast cells or accumulation of metabolites in the media leading to cloudiness during exposure. However, before sample collection, larvae were washed, and fresh media was added. The alternative explanation is that exposure to light during sampling could have induced the vision-associated genes. Prior studies have reported the expression of phototransduction pathway-associated genes (*opsin* and *rhodopsin*) in the skin of octopus (*Octopus bimaculoides*), Japanese flounder (*Paralichthys olivaceus*), Atlantic salmon (*Salmo salar*) and zebrafish (73, 74). Furthermore, expression of photoreceptor genes found in brain and other tissues of animals suggests their possible association in physiological functions, including modulation of the circadian clock (75). For instance, *vertebrate ancient-long opsin* expressed in the spinal cord of embryonic zebrafish regulates the light-mediated neural activity during embryonic development (76).

Genes from the solute carrier family such as *slc26a3.2* (chloride anion exchanger), *slc38a11* (amino acid transmembrane transporter activity) and *slc24a2* (sodium /potassium /calcium exchanger) were up-regulated in yeast-exposed GF zebrafish larvae. Modulation of these host ion channels are important to regulate cellular chemical homeostasis and they are key players in many cellular processes, including cell proliferation and cell signaling (77). The gene *tdo2a* encodes tryptophan 2,3-dioxygenase protein, which is involved mainly in tryptophan catabolism (78). A recent study has also reported the upregulation of *tdo2a* in adult zebrafish that were stimulated with β-glucans (79), suggesting that yeast exposure could regulate the tryptophan metabolism.

Interestingly, only two genes were downregulated: *ankrd37* and *egln3*. The former is involved in the modulation of protein–protein interactions (80) and the latter helps in the maintenance of oxygen homeostasis (81). We found 11 unknown genes, some of which might also be involved in host biological processes. For instance, *si:dkeyp-73b11.8* encodes serine protease inhibitor that antagonizes the biosynthesis of serine-type proteases.

There are a few previous studies in which GF zebrafish were exposed to different types of yeast and bacteria isolated from fish and mammals (8, 15, 82–86). However, these studies examined only a handful of genes to understand the host response to yeast/bacteria exposure. We have observed low expression level of immune genes (considering all the regulated genes, data not shown), which are known to be regulated in zebrafish upon experimental exposure to microbes (84, 86, 87). For example, strong or moderate induction of *il1b* was previously observed in the zebrafish larvae after exposure to bacteria or yeast (8, 84, 85), in contrast to its negligible induction in our study. Distinct host transcriptional responses against different microorganisms depend on various factors, including molecular compositions of cell wall, ability to induce immune response, route of invasion and morphotype (e.g., yeast, hyphae, and pseudohyphae forms) (88). For instance, zebrafish larvae exhibit distinct immune phagocytic responses against conidia and hyphae of the pathogenic fungi *Aspergillus fumigatus* (89).

Neutrophils are highly motile phagocytic cells from the myeloid lineage that play a critical role in triggering powerful inflammatory responses when commensal microbes exert immunological priming in most vertebrates (16). In the present study, a marked response of immune mediators was expected after *Pseudozyma* priming of GF zebrafish larvae. However, *Pseudozyma* sp. did not influence the behavior of neutrophils. Previous reports on zebrafish have also noted that fungal (*A. fumigatus* conidia) and bacterial species (*Shewanella* sp.) were unable to promote phagocytosis and the neutrophil chemotaxis, respectively, which are two key features linked to the inflammatory response in zebrafish larvae (89, 90). Emerging evidence indicates that in newly hatched zebrafish the skin keratinocytes together with neutrophils have a crucial impact on the production of inflammatory mediators (91, 92).

In the present study, we have used 3dpf larvae for the transcriptomic analysis. To rule out the possibility that the cells affected by the presence of *Pseudozyma* were the skin keratinocytes instead of the neutrophils, we examined the expression of the key inflammation markers *il1b, tnfa*, and *il10*, which were not up-regulated in yeast-treated GF larvae. As revealed by our RNA-seq data, members of the aquaglyceroporin family, ubiquitin ligase and β-galactoside-binding protein, together with the keratinocyte marker gene (*krt1-19d*) were among the immune genes up-regulated with yeast exposure. Nevertheless, the molecular mechanisms involved in the activation or enhanced expression are still not clearly understood. Our results from using a GF model demonstrate that an increase in transcript levels of metabolism-related genes correlates with the presence of the yeast in the environment. Interestingly, all species of the genus *Pseudozyma* produce mannosylerythritol (MEL) as an essential hydrophobic glycolipid with biosurfactant properties (93), which acts as an immunomodulatory and anti-tumor agent. Its anti-inflammatory activity is mediated through various pathways involving some of the proteins encoded by the genes that we found to be upregulated following yeast exposure (19, 94, 95). We did not record any inflammatory signs at the cellular or transcript levels, which suggests a plausible link between *Pseudozyma*-produced MEL and the reduced inflammatory response. However, functional studies are required to establish the mechanisms that may ultimately explain the role of *Pseudozyma* in the modulation of developmental immunity in early vertebrates. It would be also relevant to investigate how exposure to other commensal yeast species (e.g., *Debaryomyces hansenii*) influences the host transcriptome, so as to distinguish between strain-specific and general effects on the host immune system.

## Conclusions

Overall, our results indicate that priming of zebrafish larvae with *Pseudozyma* sp. can affect the host metabolic pathways, including primary bile acid biosynthesis, drug and xenobiotic metabolism, as well as some immune-related genes. However, neither key genes nor cells involved in the inflammatory response were significantly affected. We have gathered evidence that host-derived yeast can play a role in shaping host physiology. Nevertheless, a complex interaction with host-associated bacteria can also shift their functional importance in host physiology.

## Data Availability Statement

The datasets generated for this study can be found in the Sequence Read Archive (NCBI) accession number PRJNA579488.

## Ethics Statement

The animal study was reviewed and approved by Bioethical Committee of the University of Murcia (licenses #537/2011, #75/2014, and #216/2014).

## Author Contributions

PS, VK, JF, VM, and JG-V conceived and designed the study. VM, JF, and VK contributed reagents and materials for the experiments. PS, JG-V, and AD performed the laboratory work. PS, JG-V, QZ, VK, and JF analyzed the data. PS, JG-V, VK, and JF wrote the manuscript. All authors read, revised, and approved the manuscript.

## Conflict of Interest

The authors declare that the research was conducted in the absence of any commercial or financial relationships that could be construed as a potential conflict of interest.
